# Pharmacological Inhibition of Cochlear Mitochondrial Respiratory Chain Induces Secondary Inflammation in the Lateral Wall: A Potential Therapeutic Target for Sensorineural Hearing Loss

**DOI:** 10.1371/journal.pone.0090089

**Published:** 2014-03-10

**Authors:** Masato Fujioka, Yasuhide Okamoto, Seiichi Shinden, Hirotaka James Okano, Hideyuki Okano, Kaoru Ogawa, Tatsuo Matsunaga

**Affiliations:** 1 Department of Otolaryngology, Head and Neck Surgery, Keio University, School of Medicine, Shinjuku, Tokyo, Japan; 2 Department of Physiology, School of Medicine, Keio University, School of Medicine, Shinjuku, Tokyo, Japan; 3 Department of Otorhinolaryngology, Inagi Municipal Hospital, Inagi, Tokyo, Japan; 4 Department of Otolaryngology, Saiseikai Utsunomiya Hospital, Utsunomiya, Tochigi, Japan; 5 Division of Regenerative Medicine, Jikei University School of Medicine, Tokyo, Japan; 6 The Laboratory of Auditory Disorders and Division of Hearing and Balance Research, National Institute of Sensory Organs, National Tokyo Medical Center, Meguro, Tokyo, Japan; National Institutes of Health, United States of America

## Abstract

Cochlear lateral wall has recently been reported as a common site of inflammation, yet precise molecular mechanisms of the inflammatory responses remain elucidated. The present study examined the inflammatory responses in the lateral wall following acute mitochondrial dysfunction induced by a mitochondrial toxin, 3-nitropropionic acid (3-NP). Reverse-transcription (RT)-PCR revealed increases in the expression of the proinflammatory cytokines interleukin (IL)-1β and IL-6. Immunohistochemistry showed an increase in the number of activated cochlear macrophages in the lateral wall, which were in close proximity to IL-6-expressing cells. A genome-wide DNA microarray analysis of the lateral wall revealed that 35% and 60% of the genes showing >2-fold upregulation at 1 d and 3 d post-3-NP administration, respectively, were inflammatory genes, including CC- and CXC-type chemokine genes. High expression of CCL-1, 2, and 3 at 1 d, and of CCL-1, 2, 3, 4, and 5, CCR-2 and 5, and CX3CR1 at 3 d post-3-NP administration, coupled with no change in the level of CX3CL1 expression suggested that macrophages and monocytes may be involved in the inflammatory response to 3-NP-mediated injury. Quantitative (q)RT-PCR showed a transient induction of IL-1β and IL-6 expression within 24 h of 3-NP-mediated injury, followed by sustained expression of the chemoattractants, CCL-2, 4 and 5, up until 7 d after injury. The expression of CCL-2 and IL-6 was higher in animals showing permanent hearing impairment than in those showing temporary hearing impairment, suggesting that these inflammatory responses may be detrimental to hearing recovery. The present findings suggest that acute mitochondrial dysfunction induces secondary inflammatory responses in the lateral wall of the cochlear and that the IL-6/CCL-2 inflammatory pathway is involved in monocyte activation. Therefore, these secondary inflammatory responses may be a potential post-insult therapeutic target for treatments aimed at preventing the damage caused by acute mitochondrial dysfunction in the cochlear lateral wall.

## Introduction

Mitochondrial dysfunction in the cochlea is a well-known cause of sensorineural hearing loss. Mutations in mitochondrial DNA cause both syndromic and nonsyndromic deafness, and the inner ear is considered highly susceptible to mitochondrial dysfunction [5], [11], [19]. Recently, we established a novel rat model of acute mitochondrial dysfunction in the cochlea by applying 3-nitropropionic acid (3-NP) directly to the round window membrane [10]. 3-NP irreversibly inhibits the mitochondrial complex II enzyme, succinate dehydrogenase, by blocking the mitochondrial electron transport chain [1], [3]. Using the aforementioned model, primary histological changes were detected in the lateral wall spiral ligament with a degeneration of its mitochondria, in which the endocochlear potential is produced [17]. Treatment with 300 mM 3-NP resulted in temporary hearing loss (temporary threshold shift (TTS) model), whereas treatment with 500 mM 3-NP resulted in profound and permanent hearing loss (permanent threshold shift (PTS) model) [10], [17]. Because local ATP deprivation in the inner ear results from inhibition of inner ear mitochondrial function. this model replicates the etiology of inner ear energy failure caused by ATP deprivation due to inner ear ischemia. It has been reported that inner ear ischemia that results from occlusion of the anterior inferior cerebellar artery causes sensorineural hearing loss [14], [24]. In addition, circulatory disturbances (most often vertebrobasilar ischemia) and inflammation (most often viral) are the most common etiologies in sudden deafness [8], [16].

3-NP blocks cellular energy production by disrupting the mitochondrial electron transport chain. Therefore, we speculated that the primary pathophysiology in the rat model was apoptosis. Indeed, systemic application of a pan-caspase inhibitor (Z-VAD-FMK) prior to 3-NP treatment reduces the number of apoptotic cells in the lateral wall and significantly ameliorates hearing impairment [15]. However, this effect is observed only when the inhibitor is administered prior to the insult. The results suggest that apoptosis occurs at the early pathophysiological stage, and that a secondary event occurs at a later stage; this secondary event may be a potential therapeutic target after the onset of deafness.

Because the damaged spiral ligament is a common site of inflammation induced by various types of insult, including surgical stress, noise exposure, and immune-mediated treatments [6], [9], [18], [21], the present study examined the inflammatory responses following acute mitochondrial dysfunction in the cochlea using both TTS and PTS models induced by treatment with 3-NP. IL-6 is a cytokine that is critical for the recruitment of inflammatory cells. The expression of IL-6 and other proinflammatory cytokines was detected in the lateral wall, along with prominent infiltration of the cochlear macrophages adjacent to the IL-6-expressing type III fibrocytes by cochlear macrophages. Genome-wide microarray analyses of the lateral wall revealed upregulation of numerous inflammatory genes related to the infiltration of monocytes and to the activation of macrophages. qRT-PCR showed a transient induction in the expression of IL-1β and IL-6 within 24 h of 3-NP administration, followed by sustained expression of the chemoattractants, CCL-2, 4 and 5, up until 7 d after 3-NP administration. The IL-6/CCL-2 signaling pathway was activated in both the PTS and TTS models, although the levels of activation were higher in the PTS model. The present findings show that inflammatory responses play an important role in the mitochondrial dysfunction observed in the cochlear lateral wall. Differences in the inflammatory responses between the PTS and TTS models suggest that the secondary inflammatory responses may be a potential therapeutic target, leading to a treatment for deafness caused by cochlear energy failure.

## Results

### 1. Expression of proinflammatory cytokines in the 3-NP-injured cochlea

**Figure 1:**
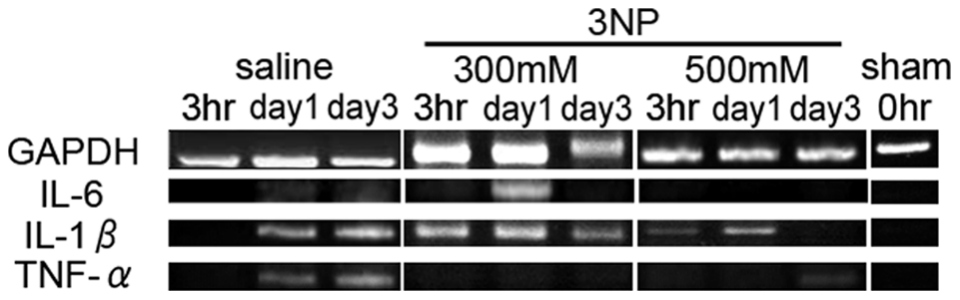
Expression of proinflammatory cytokines in 3-NP-injured cochlea. RT-PCR data for three major proinflammatory cytokines, IL-6, IL-1β, and TNF-α, and the house-keeping gene GAPDH. There was a significant difference in IL-6 expression in the 3-NP-treated cochleae compared with the saline-treated controls. Whole cochleae were harvested and used for the assay.

To detect inflammation in the 3-NP-injured cochlea, we measured the expression of TNF-α, IL-1α, IL-1β, IL-1RA, IL-6 and IL-12 p40 mRNA (all proinflammatory cytokines) using RT-PCR. We observed a temporal upregulation in IL-6 expression 1 d after 3-NP treatment; no such increase was noted in the controls. IL-1β was expressed in both 3-NP-injured and saline-treated cochlea; however, TNF-α was downregulated after treatment with 3-NP (Fig. 1). No expression of IL-1α, IL-1RA or IL-12 p40 mRNA was detected (data not shown). Whole cochlea were harvested from each animal for use in the assay.

### 2. Quantitative analysis of IL-6 expression in the cochlea

**Figure 2:**
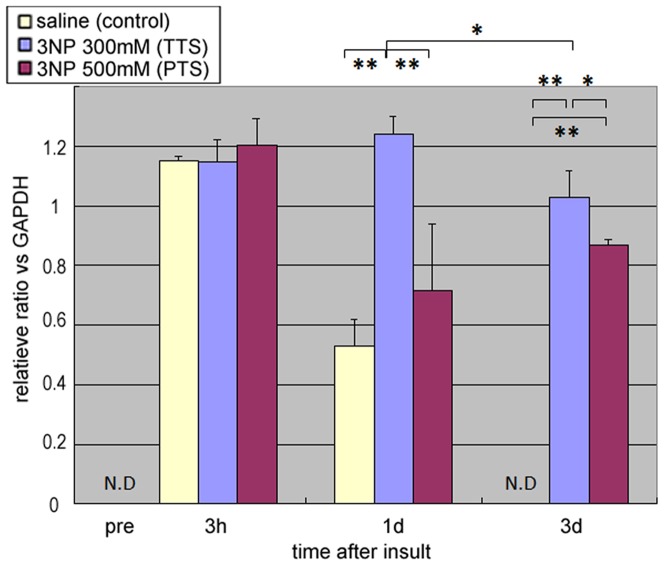
IL-6 expression in 3-NP-injured cochlea. Expression of IL-6 mRNA in whole cochlea. The temporal threshold shift (TTS) model (induced with 300 mM 3-NP) showed a significant induction of IL-6 mRNA from Day 1, which was sustained up until Day 3. The expression of IL-6 mRNA at Day 3 was also significantly higher in the permanent threshold shift (PTS) model (induced by 500 mM 3-NP) than in the saline-treated controls (*p<0.01, **p<0.05; N.D: not detected).

Time-dependent changes in IL-6 mRNA expression were quantified by qRT-PCR. In the TTS model (induced with 300 mM of 3-NP), significant expression of IL-6 was observed from Day 1 (P<0.01) and was maintained up until Day 3 (P<0.01). Expression of IL-6 was also observed in the saline-treated controls from 3 h post-surgery and it gradually decreased to undetectable levels by Day 3. Similar levels of IL-6 expression were observed in all three groups, saline control, TTS and PTS models, at 3 h post-surgery. In the PTS model (induced with 500 mM of 3-NP), IL-6 expression increased at 3 h post-3-NP administration but decreased to the saline-treated control level at Day 1; weak expression was sustained until Day 3 (Fig. 2). Note that the expression was not detected in the cochlea without any treatment or pre-treatment. Whole cochleae were used for the assays.

### 3. Emergence of cochlear macrophages in the damaged lateral wall, adjacent to the IL-6 expressing cells

**Figure 3:**
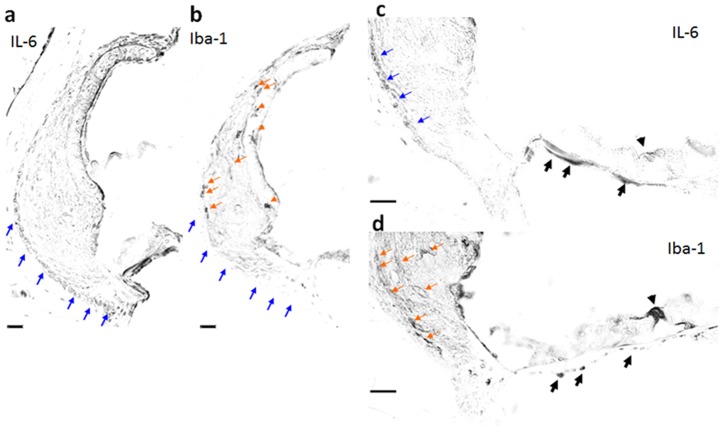
IL-6-expressing cells in 3-NP-injured cochlea were accompanied by macrophages. Cells expressing IL-6 (**a** and **c**) or Iba-1 (**b** and **d**) in the temporal threshold shift (TTS) model were examined by immunohistochemistry at Day 1 by using neighboring sections. IL-6 was expressed in type III fibrocytes (**a** and **c**; blue arrows) and in the mesenchyme cells beneath the basement membrane (**c**; black arrows). Cochlear macrophages expressing Iba-1 were identified adjacent to cells expressing IL-6, including in the lateral portion of the type II fibrocyte region that contacts type III cells (**b** and **d**; orange arrows), and were seen infiltrating the cells beneath the mesenchyme of the basilar membrane (**d**; black arrows). Iba-1-expressing cells were not observed in the type III area in which IL-6 was expressed (blue arrows in Fig. 3**a** and **b**).

We next performed immunohistochemical analyses to examine the expression of IL-6 (Fig. 3**a and c**) and the cochlear macrophage marker, Iba-1 (Fig. 3**b and d**), in the TTS model at Day 1 (when the induction of IL-6 was maximal). IL-6 was expressed by type III fibrocytes (Fig. 3**a and c**; blue arrows) and by the mesenchyme cells beneath the basement membrane (Fig. 3**c**, black arrows). Iba-1-expressing cochlear macrophages were observed in the same areas as type II fibrocytes, and were most frequently observed on the lateral side, adjacent to the IL-6-expressing type III cells (Fig. 3**b and d**; orange arrows); however, they were not observed in the same area as type III fibrocytes (blue arrows in Fig. 3**a and b**). Many Iba-1-expressing cells in the cochlea lateral wall had cellular processes, indicating that the macrophages had been activated. Iba-1-positive cells were also observed beneath the mesenchyme of the basilar membrane, but these cells did not have cellular processes (Fig. 3**d**, black arrows).

### 4. Genome-wide gene expression profiling of the lateral wall spiral ligament

Immunostaining revealed an inflammatory response to 3-NP in the cochlear lateral wall, particularly in the apical side of the basal turn. The molecular mechanism(s) underlying this inflammatory reaction remains to be elucidated. The spiral ligaments of the upper basal turns of the 3-NP-injured cochlea were harvested 1 d and 3 d post-administration of either 300 mM of 3-NP or saline, and subjected to genome-wide gene expression profiling (GEO accession number: 02708065). In the TTS model, 35% (26 of 74) and 60% (18 of 30) of the genes upregulated at Day 1 and Day 3 post-3-NP administration, respectively, were inflammatory genes, including CXC-type chemokines (Table 1) and CC-type chemokines (Table 2). Both types of chemokine induce chemotaxis by binding to their respective receptors, which are expressed on the surfaces of their target immune cells. We found high expression of CCL-1, 2 and 3 at Day 1, and high expression of CCL-1, 2, 3, 4, and 5, CCR-2 and 5, and CX3CR1 at Day 3 post-3-NP administration; however, there was no change in the expression of CX3CL1 (Tables 1 and 2). CCR-2 is an essential receptor for chemotaxis and is abundantly expressed on the surface of monocytes. CCL-2, a chemokine ligand for CCR-2, was upregulated in the 3-NP-injured lateral wall (Table 2). IL-1β and IL-6 were also detected. A>2-fold increase in gene expression compared with that in the saline-treated control was used as the cut-off threshold.

**Table 1:** Upregulated CXC-type chemokine genes in the lateral wall of 3-NP-injured cochlea.

Receptor and its cell types	Chemokine Ligands	Day1	Day3
CXCR2 (not detected)	CXCL1	7	2.9
neutrophils	CXCL2	3.5	2.2
monocytes	CXCL3	NC	NC
	CXCL5	NC	NC
CXCR3 (not detected)	CXCL9	8.2	4.1
various cell types	CXCL10	6.5	NC
	CXCL11	6.3	NC
CXCR4	CXCL12	2	NC
widely expressed			
CX3CR1	CX3CL1	NC	NC
macrophages			

The values displayed refer to the -fold changes compared with the saline-treated controls.

Genes showing >2-fold changes are listed.

NC: no change compared with control.

**Table 2:** Upregulated CC-type chemokine genes in the lateral wall of 3-NP-injured cochlea.

Receptor and its cell types	Chemokine ligands	Day 1	Day 3
CCR2	CCL2	6.6	3.5
monocytes	CCL7	NC	NC
dendritic cells	CCL8	NC	NC
	CCL13	NC	NC
CCR5	CCL3	3.8	2.1
monocytes	CCL4	NC	2.3
T lymphcyte	CCL5	NC	2.6

The values displayed refer to the -fold changes compared with the saline-treated controls.

Genes showing >2-fold changes are listed.

NC: no change compared with control.

### 5. Acute IL-6 induction and sustained chemokine expression in the damaged cochlea lateral wall

**Figure 4:**
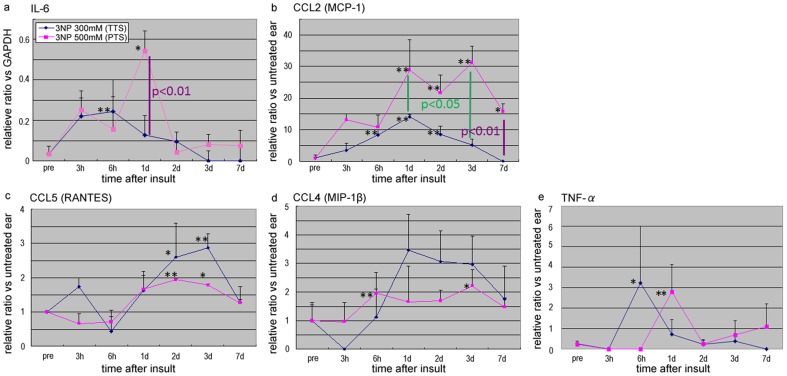
Time-dependent expression of IL-6 and chemokines in the 3-NP-injured cochlea lateral wall. Quantitative RT-PCR to examine the expression of IL-6 and chemokines, including CCL2 (MCP-1), CCL5 (RANTES) and CCL4 (MIP-1β), in the injured cochlear lateral wall. The expression of IL-6 was induced 3 h after 3-NP administration. IL-6 expression was higher in the PTS model (blue lines) than in the temporal threshold shift model (pink lines) at Day 1 (**a**; p<0.01; purple), suggesting that IL-6 was a detrimental factor. IL-6 expression was high in the PTS model at Day 1 post-injury but was quickly downregulated. Chemokines were induced in the 3-NP-injured lateral wall from 6 h to 7 d post 3-NP administration. Chemokine expression was induced more slowly than that of proinflammatory cytokines, but it was sustained (**a**–**d**). The level of CCL2 expression in the PTS and TTS models was significantly different, being greater in the PTS model than in the TTS model (**b**; p<0.05 (green) and p<0.01 (purple)), suggesting that CCL2 was also a detrimental factor. * indicates p<0.05, ** indicates p<0.01.

Next, time-dependent changes in the expression of proinflammatory genes, chemokines, and chemoattractants in the lateral wall were examined by qRT-PCR. IL-6 was significantly upregulated in both the TTS and PTS models, before being rapidly downregulated to normal levels (Fig. 4**a**: p<0.01). The peak level of IL-6 expression was significantly higher in the PTS model than in the TTS model (Fig. 4**a**; p<0.01). Chemokines and chemoattractants, including the CCL and/or the CXCL families, play an essential role in recruiting inflammatory cells to the peripheral tissues. Many of these are induced by proinflammatory cytokines such as IL-6 (and were also detected in the genome-wide assay; Tables 1 and 2). Thus, we next examined the expression of several CCL-type chemokines, including CCL-2 (MCP-1), CCL-5 (RANTES) and CCL-4 (MIP-1β). All of these chemokines were expressed in the lateral wall of the cochlea from 6 h to 7 d after 3-NP administration, and their expression was sustained for longer than that of IL-6, which was upregulated at the early stages and then quickly downregulated in a time-dependent manner (Fig. 4**a–d**). The levels of CCL-2 in the PTS and TTS models were significantly different, being greater in the PTS model than in the TTS model (Fig. 4**b**; p<0.01). We also found the expression of TNF-α but was not as much induced as IL-6 (Fig. 4**e**).

## Discussion

The pathology in the model of acute mitochondrial dysfunction described in the present study begins with the degeneration of the lateral wall of the cochlear. Fibrocytes maintain the homeostatic circulation of ions and the endocochlear potential of the lateral wall, whereas mitochondria integrate the apoptotic pathways and the production of reactive oxygen species, which cause cell damage [7]. Both apoptosis and the formation of reactive oxygen species are involved in the pathophysiological mechanisms underlying ischemia-, ototoxin-, and noise-induced cochlear damage [12], [22], [23]. Thus, mitochondrial dysfunction is likely to play a critical role in hearing loss. One critical feature of the model used in this study is that infusion of a lower concentration of 3-NP leads to TTS, despite the fact that the drug irreversibly blocks mitochondrial complex II. This suggests that there may be cellular and/or intra-cellular sources that may help the recovery of injured fibrocytes. A comparison between the TTS and PTS models may be a useful strategy in the search for potential therapies.

The inner ear had been believed as an “immune-privileged organ” and it is true that sensorineural hearing loss models show no evidence of neutrophil infiltration in the absence of complete destruction of the cochlear structure. However, recent studies of several different cochlear damage models show the presence of other types of inflammatory cell in the injured cochlear lateral wall. Hirose and colleagues reported the infiltration of mononuclear cells into cochleae subjected to noise-induced damaged, as demonstrated by immunostaining for the leukocyte common antigen, CD45 [9]. Based on the observation of phagocytic macrophages, they claimed that the infiltrating marrow-derived cells may have “cleared” the dead cells that resulted from noise [9]. Schulte and colleagues showed that marrow-derived cells or hematopoietic stem cells could migrate and differentiate into cochlear lateral wall cells, including sodium/potassium/chloride co-transporter (NKCC)- or Na/K-ATPase-positive fibrocytes, in normal adult rodents [13]. Okano and colleagues showed that cells migrating from the bone marrow constitutively resided in the cochlea and could be identified by staining for macrophage markers such as Iba-1 [18]. They also used an elegant chimeric assay to show that the Iba-1-positive cells were continuously resupplied from the bone marrow [18]. Although all three of these groups showed the migration of circulating hematopoietic cells or marrow-derived cells into the cochlea, the mechanisms underlying this phenomenon were not examined. The present study showed that the expression of inflammatory genes, including those for chemokines and chemoattractants, was induced in the damaged lateral wall of the cochlea in comparison with the saline- treated control. These genes were expressed in the areas in which infiltrating cells are most frequently observed [9], [13], [18]. Therefore, we speculate that the intrinsic program that controls gene expression in the lateral wall cells provides a mechanism for the infiltration of inflammatory cells.

Based on our microarray analyses, it is also true that the infiltration of neutrophils is lacking, as neutrophil-specific markers, such as CXCR2, could not be detected, while their ligands, CXCL1 and 2, were induced in the damaged lateral wall (Table 1). By contrast, many other chemokines, and the receptors for other types of immune cells, were detected. The monocyte receptors CCR-2 and 5 were highly expressed, and their ligands (CCL-2 and CCL-3, 4, and 5, respectively) were upregulated after 3-NP administration. mRNA for the cochlear macrophage receptor CX3CR1 was also detected, which is consistent with the immunostaining data showing the presence of Iba-1 positive cells; however, its ligand, CX3CL1, was not induced at Day 1 or 3 post-3-NP administration. These results suggest that macrophages were not recruited from the peripheral blood via CS3CL1/CXC3CR1-dependent chemotaxis, but were derived from their precursors or from monocytes, which were recruited by the CCR/CCL pathway and activated by cytokines produced by cells within the cochlear lateral wall.

The present study showed that IL-6 was expressed by type III fibrocytes, and that immune cells were most frequently observed in-between type III and type II fibrocytes. Interestingly, a similar pattern of distribution was seen in noise-induced damaged cochlea, in which IL-6 was expressed by type IV fibrocytes and immune cells were most frequently observed in between type III and type IV fibrocytes [6]. The findings in the two different models examined in the present study suggest that the induction of IL-6 in the lateral wall at the early stages of damage determines the inflammatory responses that occur in the lateral wall. We also found that CCL-2 (MCP-1) was induced upon IL-6 production. IL-6 is a strong inducer of chemokines or chemoattractants, such as CCL-2, which recruits inflammatory cells from the peripheral blood to the local injured area via its receptor, CCR-2. CCL-2 was the only CCR-2 ligand identified in the assay. Finally, both IL-6 and CCL-2 were expressed at higher levels in the permanent hearing loss model than in the temporary hearing loss model. These results lead us to believe that the IL-6/CCL-2 pathway contributes to the inflammatory response initiated by 3-NP-induced damage to the cochlear lateral wall, and that the pathway is essential for the inflammatory reactions observed in this model. Therefore, manipulating this pathway would be a feasible approach to controlling cochlear inflammation. Further studies of this pathway should incorporate genetically-modified rodents to investigate these genes using ‘loss of function’ and ‘gain of function’ approaches.

It is widely accepted that excessive inflammation is harmful, and that blocking of the associated inflammatory responses is a potential therapeutic strategy. In the present model, the expression of IL-6 and CCL-2 in the lateral wall was significantly higher in the PTS model than in the TTS model, suggesting that excessive production of inflammatory cytokines and chemokines was detrimental to organ function. It is also widely known that an appropriate inflammatory response is essential for tissue recovery. Our previous studies showed that type II and IV fibrocytes in the spiral ligament divide after injury, and exogenous mesenchymal stem cell transplantation studies show that these cells promote fibrocyte proliferation both *in vivo* and *in vitro* [20]. CCL-type chemokines, such as CCL-2, are potent agents that recruit these cells [2], [4]. Thus, in this context, appropriate activation of the local IL-6/CCL-2 pathway may contribute to the recovery of the lateral wall of the cochlea. The results presented herein indicate that treatment with anti-inflammatory agents would be a feasible strategy for treating acute energy failure in the cochlea; however, considerable thought and pre-clinical investigation is required to identify suitable regimens, along with appropriate timings and doses.

## Conclusion

In conclusion, the present study shows that secondary inflammation occurs in the lateral wall of the cochlea after 3-NP-induced acute energy failure. Macrophage activation and the induction of inflammatory genes were detected following mitochondrial dysfunction. The expression of IL-6 and CCL-2 was higher in the permanent hearing damage model than in the temporary hearing damage model, suggesting that the inflammatory response is a potential therapeutic target for the treatment of deafness resulting from energy failure in the lateral cochlear wall. Further studies that focus on the inflammatory response in this organ will help us to understand the pathophysiology of the lateral wall damage involved in acute hearing loss.

## Materials and Methods

### 1. Animal models

All experimental procedures described in this study were approved by the Institutional Animal Care and Use Committee of the National Tokyo Medical Center, in accordance with the Guide for the Care and Use of Laboratory Animals (National Institute of Health, Bethesda, MD). Female Sprague-Dawley rats weighing between 180 g and 210 g (8–10 weeks old) were used for the experiments. The surgical protocols have been described previously [10], [17]. Briefly, after general anesthesia with pentobarbital (30–40 mg/kg, i.p.) and the local administration of lidocaine (1%), an incision was made posterior to the left pinna near the external meatus. The left otic bulla was opened and the round window niche was infused with 3-NP (500 mM or 300 mM; Sigma, St. Louis, MO, USA) dissolved in saline (pH adjusted to 7.4 with NaOH). Infusion with saline alone was used as a control. Following treatment, and before the wound was closed, a small piece of gelatin was placed onto the niche to keep the solution in place and to allow for head movement after the animals awoke. The right cochlea was surgically destroyed to avoid cross-hearing during the recording of auditory brain-stem responses. To confirm deafness, auditory brain-stem responses were recorded before and 3 h after 3-NP or saline administration, or at the end of the observation period (1, 7, and 14 d; Table S1).

### 2. RT-PCR, quantitative RT-PCR and DNA array analyses

Either the whole cochlea or the second turn of the lateral wall (excluding the stria vascularis) was harvested and reverse transcription was performed with an oligo-dT primer and a SuperScript II RT-PCR kit (Invitrogen) according to the manufacturer's protocol. The obtained cDNAs were used for RT-PCR (for whole cochlear samples taken 3 h, 1 d, and 3 d after the administration of 300 mM and 500 mM of 3-NP; n = 4 per group or samples without any treatment; n = 6) or qRT-PCR (for lateral wall samples taken before administration and 3 h, 6 h, 1 d, 2 d, 3 d, and 7 d after administration of 300 mM and 500 mM 3-NP; n = 3 per group). RT-PCR was performed over 37 cycles with primers supplied in the message Screen Rat Inflammatory Cytokine Set 2 Multiplex PCR Kits (BioSource International Inc., Camarillo, CA). The thermal cycling conditions for the Taq polymerase (Takara Biotechnology Co., Ltd., Otsu, Japan) were as stated in the manufacturer's protocol. Quantitative real-time RT-PCR reactions were carried out in a Mx3000p (Stratagene, La Jolla, CA) using FAM-conjugated TaqMan primers against IL-1β, IL-6, CCL-2, CCL-4, CCL-5 and SOCS-3, and a VIC-conjugated glyceraldehyde-3-phosphate dehydrogenase (GAPDH) probe as an internal control (Applied Biosystems, Foster City, CA). As a reference, the fluorescent dye, ROX, was used to calibrate inter-sample variability. The relative expression levels of each cytokine were statistically compared across both groups (PTS and TTS) using a one-way analysis of variance (ANOVA). Repeated measures ANOVA was used to analyze differences in the results obtained pre- and at 3 h, 6 h, 1 d, 2 d, 3 d, and 7 d post-3-NP administration (within-subject variability) and the differences between the PTS and TTS groups (between subject variability). All procedures incorporated controls that did not undergo RT or lacked the PCR template. DNA array analyses were performed using samples obtained 1 d and 3 d after 3-NP or saline administration (n = 4) and the GeneChip Rat Expression Set 230 (AFFYMETRIX). All experiments were conducted using a GeneChip Fluidics Station 400 (AFFYMETRIX), a GeneChip hybridization Oven 640 (AFFYMETRIX), and a Gene array Scanner (Agilent). Raw data and its information was uploaded to the NIH database, GEO (GEO accession number: 02708065). The data were analyzed with Signet Viewer (Biomatrix). For each gene, the ratio of the signal in the TTS sample to that in the saline-treated control was calculated and the data were analyzed.

### 3. Histology

Histological analysis was performed 1 day after the administration of 300 mM 3-NP (n = 3). For tissue fixation, rats were deeply anesthetized with pentobarbital (as described above) and transcardially perfused with 4% paraformaldehyde. Paraffin sections were prepared as previously described [20]. Immunohistochemistry for Iba-1 and IL-6 was performed as previously described [6]. All slides were washed with PBS, incubated in 1.5% hydrogen peroxide for 15 min, rinsed three times in PBS, incubated with 10% normal goat serum for 1 h at room temperature, and then incubated overnight with the appropriate primary antibodies at 48°C. The primary antibodies used were a rabbit anti-IL-6 polyclonal antibody (diluted 1∶150; Sigma) and a rabbit anti-Iba-1 polyclonal antibody (diluted 1∶500; Wako Pure Chemicals). The slides were then incubated with biotinylated secondary antibodies (1∶1000) at 37°C for 30 min, washed three times in PBS, and then incubated in VECTASTAIN Elite ABC reagent (Elite ABC kit, Vector Laboratories, Burlingame, CA) for 30 min at room temperature. After gentle washing in PBS (three times), staining was visualized by incubating the samples with diaminobenzidine solution (Wako Pure Chemical Industries). After a final wash with PBS, the samples were dehydrated and mounted under coverslips.

## Supporting Information

Table S1 Hearing thresholds of the animals used for the RT-PCR experiments. Eight and twenty kilohertz tone burst auditory brain-stem responses were measured at the indicated time-points (dBSPL). Note that all animals suffered consistent hearing impairment.
(DOCX)
