# Supplementary material for: Pharmacological Inhibition of Cochlear Mitochondrial Respiratory Chain Induces Secondary Inflammation in the Lateral Wall: A Potential Therapeutic Target for Sensorineural Hearing Loss
Source: PLoS One. 2014 Mar 10;9(3):e90089. doi: 10.1371/journal.pone.0090089 (PMC3948682; doi:10.1371/journal.pone.0090089)
Supplement: Supplementary file 1 [file pone.0090089.s001.docx]

	8kHz							20kHz						
	pre	3 hr	6 hr	1 d	2d	3d	7d	pre	3 hr	6 hr	1 d	2d	3d	7d
300mM 3hr- ① ○	2	87						1	86					
300mM 3hr- ② ○	2	62						1	81					
300mM 3hr- ③ ○	12	42						6	66					
300mM ①hr- ○ ▯	2	62	62					1	66	66				
300mM 6hr- ③ ○	2	57	62					1	66	71				
300mM 6hr- ④ ○	2	57	57					1	61	71				
300mM ⑤d- ○ ▯	7	57		32				1	71		86			
300mM 1d- ⑥ ○	2	47		47				1	76		86			
300mM 1d- ⑧ ○	7	67		62				1	71		91			
300mM ④d- ○ ▯	7	92		92	87			6	91		91	76		
300mM 2d- ⑤ ○	2			62	37			1			91	81		
300mM 2d- ⑥ ○	7	72		62	32			6	71		91	81		
300mM ⑤d- ○ ▯	7			72	72	57		6			76	91	81	
300mM 3d- ⑥ ○	7			67	27	12		1			91	81	76	
300mM 3d- ⑨ ○	2			92	92	82		6			91	81	81	
300mM ①d- ○ ▯	7			77	67	7	12	- 4			81	86	61	41
300mM 7d- ② ○	- 3			92	52	27	17	1			91	81	81	51
300mM 7d- ⑦ ○	- 3			87	92		12	1			91	91		66
	8kHz							20kHz						
	pre	3 hr	6 hr	1 d	2d	3d	7d	pre	3 hr	6 hr	1 d	2d	3d	7d
500mM 3hr- ① ○	12	87						6	91					
500mM 3hr- ② ○	2	92						1	91					
500mM 3hr- ③ ○	2	92						1	91					
500mM 6hr- ① ○	7	92	92					1	91	91				
500mM 6hr- ② ○	2	92	92					6	86	81				
500mM 6hr- ④ ○	7	92	92					1	91	86				
500mM 1d- ② ○	2	87		92				1	91		86			
500mM 1d- ③ ○	2	92		92				- 4	76		86			
500mM 1d- ④ ○	7			67				1			91			
500mM 2d- ① ○	7	72			77			1	81			91		
500mM 2d- ③ ○	7	92		92	92			6	91		81	86		
500mM 2d- ④ ○	2	92		92	92			6	81		71	91		
500mM 3d- ① ○	2			87		92		6			86		81	
500mM 3d- ③ ○	2	92		92	92	92		1	91		81	81	91	
500mM 3d- ④ ○	7			92	92	87		1			76	81	86	
500mM 7d- ① ○	7			92	92	92	82	6			91	91	91	91
500mM 7d- ② ○	- 3			92	92	92	92	1			91	91	91	91
500mM 7d- ③ ○	2						92	1						91
